# Direct role of FLT3 in regulation of early lymphoid progenitors

**DOI:** 10.1111/bjh.15578

**Published:** 2018-09-14

**Authors:** Alya Zriwil, Charlotta Böiers, Trine A. Kristiansen, Lilian Wittmann, Joan Yuan, Claus Nerlov, Ewa Sitnicka, Sten E. W. Jacobsen

**Affiliations:** ^1^ Lund Center for Stem Cell Biology and Cell Therapy Lund University Lund Sweden; ^2^ Division of Molecular Haematology Department of Laboratory Medicine Lund University Lund Sweden; ^3^ Molecular Medicine and Gene Therapy Lund Stem Cell Center Lund University Lund Sweden; ^4^ MRC Molecular Haematology Unit Weatherall Institute of Molecular Medicine John Radcliffe Hospital University of Oxford Oxford United Kingdom; ^5^ Wallenberg Institute for Regenerative Medicine Department of Cell and Molecular Biology Center for Haematology and Regenerative Medicine Department of Medicine Huddinge Karolinska Institutet and Karolinska University Hospital Huddinge Stockholm Sweden

**Keywords:** conditional knock‐out mouse model, FLT3, haematopoiesis, lymphoid progenitors, lymphoid development

## Abstract

Given that FLT3 expression is highly restricted on lymphoid progenitors, it is possible that the established role of FLT3 in the regulation of B and T lymphopoiesis reflects its high expression and role in regulation of lymphoid‐primed multipotent progenitors (LMPPs) or common lymphoid progenitors (CLPs). We generated a *Flt3* conditional knock‐out (*Flt3* ^*fl/fl*^) mouse model to address the direct role of FLT3 in regulation of lymphoid‐restricted progenitors, subsequent to turning on *Rag1* expression, as well as potentially ontogeny‐specific roles in B and T lymphopoiesis. Our studies establish a prominent and direct role of FLT3, independently of the established role of FLT3 in regulation of LMPPs and CLPs, in regulation of fetal as well as adult early B cell progenitors, and the early thymic progenitors (ETPs) in adult mice but not in the fetus. Our findings highlight the potential benefit of targeting poor prognosis acute B‐cell progenitor leukaemia and ETP leukaemia with recurrent *FLT3* mutations using clinical FLT3 inhibitors.

Haematopoiesis is characterized by a very high turnover of mature blood cells of multiple lineages as well as their progenitors, a process partly regulated by a large number of haematopoietic growth factors or cytokines (Metcalf, 2008). Signalling through multiple cell surface tyrosine kinase receptors, triggered through binding of their specific ligands, represents an important extrinsic regulation of distinct haematopoietic stem and progenitor cells both in human and mouse (Ullrich & Schlessinger, 1990; Scheijen & Griffin, 2002).

The FMS‐like tyrosine kinase 3 receptor (encoded by the *Flt3* gene, also called *Flk2*) is a type III receptor tyrosine kinase (Matthews *et al*, 1991; Rosnet *et al*, 1991; Rosnet *et al*, 1993). Its ligand, FLT3 ligand (FLT3L) exists in a soluble as well as membrane‐bound form (Lyman *et al*, 1995; Lyman & Jacobsen, 1998). Studies in mice have established that FLT3 and FLT3L play an important role in lymphopoiesis (Lyman & Jacobsen, 1998; McKenna *et al*, 2000; Sitnicka *et al*, 2002; Tsapogas *et al*, 2017). Although not expressed on haematopoietic stem cells (HSCs), FLT3 expression is initiated on multipotent progenitors (MPPs) and sustained on common lymphoid progenitors (CLPs), but is only expressed on the very earliest B‐cell and T‐cell progenitors (Wasserman *et al*, 1995; Adolfsson *et al*, 2001, 2005; Sitnicka *et al*, 2002; Boyer *et al*, 2011; Buza‐Vidas *et al*, 2011; Luc *et al*, 2012). Lymphoid‐primed multipotent progenitors (LMPPs) express the highest levels of FLT3 (Adolfsson *et al*, 2005), and FLT3 plays a key role in LMPP and CLP maintenance (Sitnicka *et al*, 2002, 2007). As recently highlighted (Tsapogas *et al*, 2017), because no *Flt3* conditional knockout mouse has been generated, it remains unclear to what degree the reductions observed in B lymphocyte and thymocyte progenitors in mice with germ line deletion of FLT3 or FLT3L (Mackarehtschian *et al*, 1995; Sitnicka *et al*, 2003, 2007), are secondary to loss of LMPPs and/or CLPs prior to becoming programmed for lymphoid‐restricted development, or also reflect a distinct role of FLT3 also in already lymphoid‐restricted progenitors. In fact, the expression of FLT3 in the B‐ and T‐lymphocyte lineages, is restricted to the earliest pre‐proB and early thymic progenitors (ETPs), respectively (Wasserman *et al*, 1995; Mansson *et al*, 2010; Luc *et al*, 2012), progenitors suggested largely to represent not fully lymphoid‐restricted progenitors (Rumfelt *et al*, 2006; Luc *et al*, 2012). Establishing to what degree FLT3 plays a direct role in regulating already lymphoid‐programmed progenitors, is of particular relevance for the high prevalence of two types of *FLT3* driver mutations, internal tandem duplication (ITD) and recurrent FLT3 point‐mutations, both associated with a poor clinical outcome in acute leukaemia (Stirewalt & Radich, 2003; Tsapogas *et al*, 2017), including distinct ETP and B‐cell progenitor leukaemia (Armstrong *et al*, 2004; Neumann *et al*, 2013).

Furthermore, cytokine receptors and their ligands are thought to play distinct roles at different stages of development, and this has been also specifically suggested for FLT3 and FLT3L (Vosshenrich *et al*, 2003; Boiers *et al*, 2013; Beaudin *et al*, 2016). To more specifically address progenitor stage‐ and ontogeny‐specific roles of FLT3 in regulation of normal lymphopoiesis, we generated a *Flt3 floxed/floxed* conditional knock‐out (*Flt3* ^*fl/fl*^) mouse line, allowing us to specifically target FLT3 deletion in a temporal and spatial manner.

## Methods and materials

### Animals

The *Flt3 floxed/floxed* conditional knock‐out (*Flt3* ^*fl/fl*^) mouse line was generated using a DNA targeting construct in which the genomic fragment of the mouse *Flt3* gene has exon 15 flanked by LoxP sites (flox) and with an Frt‐neomycin‐Frt cassette inserted into intron 15 of the mouse *Flt3* gene. The IB10/C embryonic stem (ES) cell line (E14 subclone 129/Ola) was electroporated with the targeting construct and targeted clones selected using neomycin. Correctly‐targeted ES clones were introduced into C57BL6 blastocysts by injection into the blastocyst cavity. Injected blastocysts were then transplanted to the uterus of pseudo‐pregnant foster mothers. Offspring positive for the floxed *Flt3* allele were then crossed with Flp‐deleter mice to remove the neomycin cassette. Screening of *Flt3* ^*fl/fl*^ mice was carried out using 2 primers flanking the 5′ loxP site Primer 1: AGATGCCAGGACATCAGGAACCTG and Primer 2: ATCAGCCACACCAGACACAGAGATC. *Flt3* ^*fl/fl*^ mice were then backcrossed for more than 5 generations with C57/Bl6 mice and subsequently crossed with different Cre‐recombinase mouse strains (all on a C57/Bl6 genetic background).

*Vav1* ^*cre/+*^ *, Mx1* ^*cre/+*^, *Rag1* ^*cre/+*^ mice have been previously described (Kuhn *et al*, 1995; McCormack *et al*, 2003; Stadtfeld & Graf, 2005). For each cross, non‐Cre expressing *Flt3* ^*fl/fl*^ females were bred with *Flt3* ^*fl/fl*^ males heterozygous for the *Cre* of interest to yield *Cre* ^*+*^ *Flt3* ^*fl/fl*^ as well as *Cre* ^*−*^ *Flt3* ^*fl/fl*^ control littermates. For timed pregnancies, mice were mated late afternoon and females were checked the following morning for the presence of a vaginal plug designated as embryonic day 0·5 (E0·5).

All mice were maintained under specific pathogen‐free conditions at Lund University Animal Facility. The Ethical Committee at Lund University approved all performed experiments.

### Dissections and cell preparations

The fetal liver (FL) and fetal thymus were dissected and mechanically disrupted with a syringe. Bone marrow (BM) cells were extracted from femora and tibia using a mortar. Peritoneal cavity lavage was performed using 10 ml of phosphate‐buffered saline (PBS) (Thermo Fisher Scientific Inc, Logan, UT, USA) containing 5% of Fetal Bovine Serum (FBS) (Hyclone, Logan, UT, USA). Single‐cell suspensions were prepared in PBS containing 5% of FBS and filtered through a 70‐μm cell strainer (BD Biosciences, San Jose, CA, USA). Cells were counted with the Sysmex (KX‐21N) Haematology analyser (Sysmex Corporation Europe GmbH, Norderstedt, Germany).

### Flow cytometry and fluorescence‐activated cell sorting (FACS)

Dissected fetal tissues and adult BM were treated with purified anti‐CD16/32 antibody (Fc‐block) and then stained with specific mouse monoclonal antibodies (mAb). mAbs used to stain cell surface markers are listed in Table SI. 7‐aminoactinomycinD (7‐AAD, Sigma‐Aldrich Company Ltd, St. Louis, MO, USA) was used to exclude dead cells from the analysis. Samples were analysed on an LSRII (BD Biosciences) and analysis was performed using FlowJo software (version 9.3; TreeStar, Ashland, OR, USA). For all the flow cytometry profiles shown, singlet viable cells were first gated as lineage negative and further gating is indicated with arrows.

### Induction of *Flt3* deletion

*Mx1* ^*cre/+*^ *Flt3* ^*fl/fl*^ and *Mx1* ^*+/+*^ *Flt3* ^*fl/fl*^ mice were injected at 7 weeks with 5 intraperitoneal injections of 300 μg of polyinositolic polycytidylic acid (pIpC) at two‐day intervals. Mice were analysed at 4 weeks post‐injection. Deletion efficiency was assessed by sorting 100 000 cells, extracting DNA and performing polymerase chain reaction (PCR) using the KAPA Mouse Genotyping Kit from KAPA Biosystems (Wilmington, MA, USA) with the following primers: Primer 1: AGATGCCAGGACATCAGGAACCTG, Primer 2: ATCAGCCACACCAGACACAGAGATC and Primer 3: CAGTCCCGAGGGGA TGATAC according to the manufacturer protocol.

### Transplantation assay

Lethally irradiated (900 cGy) 12‐ to 16‐week‐old C57BL/6 CD45.1 wild type (WT) recipient mice were transplanted intravenously with 2 × 10^6^ cells unfractionated BM cells from *Mx1* ^*cre/+*^ *Flt3* ^*fl/fl*^ (CD45.2) or *Mx1* ^*+/+*^ *Flt3* ^*fl/fl*^ (CD45.2) mice together with 2 × 10^6^ unfractionated BM competitor cells from WT CD45.1 mice, or 2 × 10^6^ unfractionated E14.5 FL cells from *Rag1* ^*cre/+*^ *Flt3* ^*fl/fl*^ (CD45.2) or *Rag1* ^*+/+*^ *Flt3* ^*fl/fl*^ (CD45.2) together with 2 × 10^6^ unfractionated E14.5 FL competitor cells from WT CD45.1 mice. Four weeks after transplantation, mice transplanted with *Mx1* ^*cre/+*^ *Flt3* ^*fl/fl*^ or *Mx1* ^*+/+*^ *Flt3* ^*fl/fl*^ BM cells were injected with 5 intraperitoneal injections of 300 μg of pIpC at two‐day intervals and then analysed for reconstitution at 8 weeks post‐transplantation.

### Statistics

Prism software (GraphPad Software Inc., La Jolla, CA, USA) was used for all statistical analysis. Statistical significances were determined using an unpaired Mann–Whitney test. The significance level was set at *P* < 0·05.

## Results

### Requirement for FLT3 during adult haematopoiesis

To investigate the requirement for FLT3 at different stages of development and distinct haematopoietic progenitor stages, we generated mice in which loxP sites had been inserted into the flanking introns of exon 15 of the *Flt3* gene (*Flt3* ^*fl/fl*^). Exon 15 encodes for a kinase ATP binding domain required for signal transduction upon ligand binding after dimerization and auto‐phosphorylation of the FLT3 receptor (Ubersax & Ferrell, 2007). As such, excision of exon 15 using Cre/loxP recombination should result in a non‐functional FLT3 protein. It was however unclear to what degree this targeting strategy also would result in loss of FLT3 protein expression. Therefore, to first validate the impact of this targeting strategy on FLT3 expression and haematopoiesis, we crossed *Flt3* ^*fl/fl*^ mice with *Vav1* ^*cre/+*^ mice, which efficiently targets Cre expression to the entire haematopoietic system, including HSCs, from an early stage of haematopoietic development following emergence of definitive HSCs (Ogilvy *et al*, 1999; Almarza *et al*, 2004; Stadtfeld & Graf, 2005). Whereas bone marrow cellularity was not affected (Figure S1A), adult *Vav1* ^*cre/+*^ *Flt3* ^*fl/fl*^ mice demonstrated a complete loss of FLT3 expression on Lin^*−*^SCA‐1^+^KIT^+^ (LSK) cells as well as Lin^−^SCA‐1^low^KIT^low^IL7R^+^ CLPs (Figure S1B). Moreover, in agreement with previous studies of conventional germ‐line *Flt3* and *Flt3l* knockout mice (in which *Flt3* o*r Flt3l* expression is permanently disrupted in the entire mouse) (Mackarehtschian *et al*, 1995; McKenna *et al*, 2000; Sitnicka *et al*, 2003, 2007), pan‐haematopoietic loss of FLT3 expression from early fetal development did not affect numbers of LSKCD48^*−*^CD150^+^ long‐term (LT)‐HSCs (Figure S1C). Whereas LSKCD48^*−*^CD150^*−*^ short‐term (ST)‐HSCs/MPPs and CD48^+^CD150^+^ MPPs were also unaffected in adult *Vav1* ^*cre/+*^ *Flt3* ^*fl/fl*^ mice, distinct reductions were observed in CD48^+^CD150^*−*^MPPs which include the majority of LMPPs (Kiel *et al*, 2005; Mead *et al*, 2013), CLPs (Figure S1B–C), B‐lymphoid restricted progenitors (Figure S1D), and the earliest (double‐negative; DN) progenitors in the thymus, (Figure S1E).

**Figure 1:**
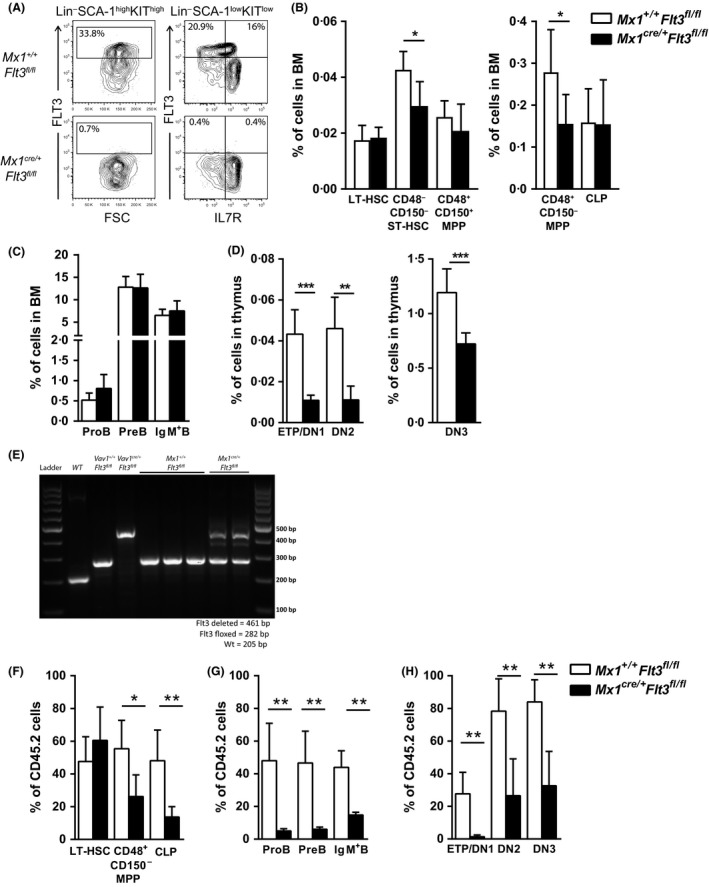
Role of FLT3 in steady‐state adult haematopoiesis. (A) Representative fluorescence‐activated cell sorting (FACS) profiles showing FLT3 surface expression on Lin^−^SCA‐1^+^KIT^+^ cells and Lin^−^SCA‐1^low^KIT^low^ cells in *Mx1* ^*+/+*^ *Flt3* ^*fl/fl*^ compared to *Mx1* ^*cre/+*^ *Flt3* ^*fl/fl*^ bone marrow (BM) (numbers represent mean percentages of 6–8 mice per genotype) 4 weeks after polyinositolic polycytidylic acid (pIpC) injection. In addition to isotype control and Fluorescence Minus One (FMO) controls, gates for FLT3 expression were set using long‐term haematopoietic stem cells (LT‐HSCs) as a negative internal reference population (IRP), to improve the reliable detection of FLT3 positive and negative cells, as HSCs have been established to lack cell surface FLT3 expression (Adolfsson *et al*, 2001). (B–C) Mean percentages (±SD of total BM cells) of (B) LT‐HSCs (Lin^−^SCA^−^1^+^KIT^+^CD48‐CD150^+^), CD48^*−*^ CD150^*−*^ short‐term (ST)‐HSCs (Lin^−^SCA‐1^+^KIT^+^CD48^−^CD150^−^), CD48^+^ CD150^+^ multipotent progenitors (MPPs) (Lin^−^SCA‐1^+^KIT^+^CD48^+^CD150^+^), CD48^+^ CD150^*−*^ MPPs (Lin^−^SCA‐1^+^KIT^+^CD48^+^CD150^−^) and common lymphoid progenitors (CLPs) (Lin^−^SCA‐1^low^KIT^low^IL‐7R^+^), (C) ProB cells (Lin^*−*^B220^+^ CD43^+^ CD19^+^ CD24^+^ CD93^+^), PreB cells (Lin^*−*^B220^+^ CD43^*−*^ CD19^+^IgM^*−*^) and IgM^+^ B cells (Lin^*−*^B220^+^ CD43^*−*^ CD19^+^IgM^+^) in 12‐week‐old *Mx1* ^*+/+*^ *Flt3* ^*fl/fl*^ and *Mx1* ^*cre/+*^ *Flt3* ^*fl/fl*^ mice (*n* = 6–8 mice per genotype in 3 experiments) 4 weeks after pIpC injection. (D) Mean percentages (±SD of total thymus cells) of early thymic progenitors (ETPs) (Lin^*−*^ CD4^*−*^ CD8a^*−*^ KIT ^+^ CD25^*−*^), Double Negative 2 (DN2) (Lin^*−*^ CD4^*−*^ CD8a^*−*^ KIT ^+^ CD25^+^) and Double Negative 3 (DN3) (Lin^*−*^ CD4^*−*^ CD8a^*−*^ KIT ^*−*^ CD25^+^) cells in 12‐week‐old *Mx1* ^*+/+*^ *Flt3* ^*fl/fl*^ and *Mx1* ^*cre/+*^ *Flt3* ^*fl/fl*^ mice (*n* = 6–8 mice per genotype in 3 experiments) 4 weeks after pIpC injection. (E) Polymerase chain reaction analysis of recombination at the *Flt3* locus in ProB cells in *Mx1* ^*+/+*^ *Flt3* ^*fl/fl*^ and *Mx1* ^*cre/+*^ *Flt3* ^*fl/fl*^ mice 4 weeks after pIpC injection. Also shown are *Vav1* ^*+/+*^ *Flt3* ^*fl/fl*^, *Vav1* ^*cre/+*^ *Flt3* ^*fl/fl*^ and wild type (WT) controls. The upper band represents the deleted Flt3 allele (461 bp), the middle band the floxed Flt3 allele (282 bp) and the lower band the WT allele (205 bp). (F–H) Mean percentages (±SD) contribution of CD45.2 cells to (F) LT‐HSCs, CD48^+^ CD150^*−*^ MPPs and CLPs, (G) ProB cells, PreB cells and IgM^+^ B cells in BM and (H) ETP, DN2 and DN3 cells in thymus of mice transplanted with 2 × 10^6^ cells unfractionated BM cells from *Mx1* ^*+/+*^ *Flt3* ^*fl/fl*^ (CD45.2) or *Mx1* ^*cre/+*^ *Flt3* ^*fl/fl*^ (CD45.2) mice (*n* = 6 per genotype) together with 2 × 10^6^ cells unfractionated BM competitor cells from WT CD45.1 mice, analysed 8 weeks post‐transplantation and 4 weeks after pIpC injection. *P < 0·05; **P < 0·01; ***P < 0·001.

As previous studies have suggested that FLT3 might have distinct roles in adult and fetal haematopoiesis (Vosshenrich *et al*, 2003; Boiers *et al*, 2013; Beaudin *et al*, 2016), we next specifically investigated the role of FLT3 in adult haematopoiesis by crossing *Flt3* ^*fl/fl*^ with *Mx1‐cre* ^*cre/+*^ mice in which Cre is only expressed upon induction with either interferon‐α or the interferon inducer pIpC (Kuhn *et al*, 1995). We treated adult (8‐week‐old) *Mx1* ^*cre/+*^ *Flt3* ^*fl/fl*^ and *Mx1* ^*+/+*^ *Flt3* ^*fl/fl*^ mice with intraperitoneal pIpC injections and analysed the impact 4 weeks later. As in *Vav1* ^*cre/+*^ *Flt3* ^*fl/fl*^ mice, FLT3 expression was almost completely lost on Lin^*−*^SCA‐1^+^KIT^+^ and CLPs in adult *Mx1* ^*cre/+*^ *Flt3* ^*fl/fl*^ mice following pIpC treatment (Fig 1A). No change was observed in total BM cellularity (Figure S2A), nor in the number of HSCs (Lin^−^SCA‐1^+^KIT^+^CD48^−^CD150^+^; Fig 1B), in agreement with the lack of FLT3 expression on mouse HSCs (Sitnicka *et al*, 2002; Buza‐Vidas *et al*, 2009; Beaudin *et al*, 2014, 2016). In contrast, CD48^+^CD150^*−*^ MPPs, containing LMPPs normally expressing the highest levels of FLT3 (Adolfsson *et al*, 2005; Mead *et al*, 2013), were distinctly reduced (Fig 1B). Notably, despite of the loss of FLT3 expression, CLP numbers were unaffected in adult *Mx1* ^*cre/+*^ *Flt3* ^*fl/fl*^ mice 4 weeks following pIpC treatment (Fig 1B), suggesting that the maintenance of adult CLPs is less dependent on FLT3 than LMPPs. Surprisingly, and in contrast to adult mice with constitutive knock‐out of FLT3 (Mackarehtschian *et al*, 1995), no changes were observed in distinct stages of B‐cell progenitors (ProB: Lin^*−*^B220^+^CD43^+^CD19^+^CD24^+^ CD93^+^; PreB: Lin^*−*^B220^+^CD43^*−*^CD19^+^IgM^*−*^; IgM^+^ B cells: Lin^*−*^B220^+^CD43^*−*^CD19^+^IgM^+^) in adult *Mx1* ^*cre/+*^ *Flt3* ^*fl/fl*^ mice following pIpC treatment (Fig 1C). In contrast, while no significant change was observed in total thymus cellularity (Figure S2B), a clear reduction was observed in the ETP (Lin^*−*^CD4^*−*^CD8a^*−*^KIT^+^CD25^*−*^), Double Negative 2 (DN2; Lin^*−*^CD4^*−*^CD8a^*−*^KIT^+^CD25^+^) and Double Negative 3 (DN3; Lin^*−*^CD4^*−*^CD8a^*−*^KIT^*−*^CD25^+^) thymocytes in adult *Mx1* ^*cre/+*^ *Flt3* ^*fl/fl*^ mice following pIpC treatment (Fig 1D), demonstrating a strict requirement for FLT3 function during steady‐state adult thymopoiesis.

Genomic PCR analysis of ProB cells purified from *Mx1* ^*cre/+*^ *Flt3* ^*fl/fl*^ BM demonstrated that the majority of *Mx1* ^*cre/+*^ *Flt3* ^*fl/fl*^ ProB cells had a remaining floxed Flt3 allele (Fig 1E) suggesting that non‐deleted (wild‐type) progenitors, such as CLPs, have a competitive advantage over *Flt3‐*deleted progenitors in sustaining adult early B cell progenitors. In agreement with this, upon transplantation of unfractionated adult BM cells from CD45.2 *Mx1* ^*+/+*^ *Flt3* ^*fl/fl*^ or *Mx1* ^*cre/+*^ *Flt3* ^*fl/fl*^ donor mice together with competitor CD45.1 BM cells into lethally‐irradiated adult CD45.1 recipients followed by pIpC treatment, we observed a consistently reduced contribution of *Mx1* ^*cre/+*^ *Flt3* ^*fl/fl*^ cells to CD48^+^CD150^*−*^ MPPs and CLPs, as well as B‐cell and T‐cell progenitors (Fig 1F–H, Figure S2C–E).

Thus, FLT3 plays an important role in sustaining multiple stages of lympho‐myeloid progenitors in adult haematopoiesis.

### Requirement for FLT3 after initiation of lymphoid lineage programme

**Figure 2:**
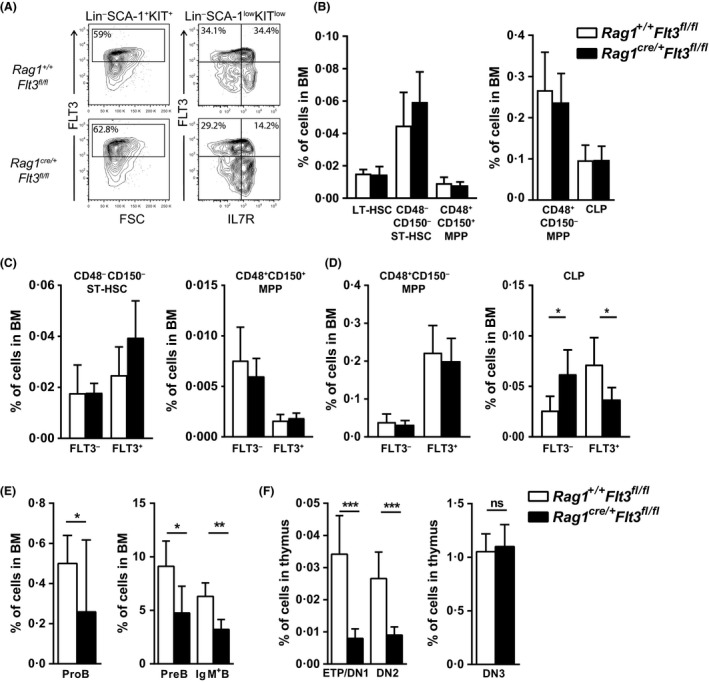
Role of FLT3 in adult lymphoid‐committed progenitors. (A) Representative fluorescence‐activated cell sorting (FACS) profiles showing FLT3 surface expression on Lin^−^SCA‐1^+^KIT^+^ cells and Lin^−^SCA‐1^low^KIT^low^ cells in *Rag1* ^*+/+*^ *Flt3* ^*fl/fl*^ compared to *Rag1* ^*cre/+*^ *Flt3* ^*fl/fl*^ bone marrow (BM) (numbers represent mean percentages of 7 mice per genotype). Gates were set using long‐term haematopoietic stem cells (LT‐HSCs) as a negative internal reference population (IRP). (B) Mean percentages (±SD of total BM cells) of CD48^*−*^ CD150^+^ LT‐HSCs, CD48^*−*^ CD150^*−*^ short‐term (ST)‐HSCs, CD48^+^ CD150^+^ multipotent progenitors (MPPs), CD48^+^ CD150^*−*^ MPPs and common lymphoid progenitors (CLPs) in 12‐week‐old *Rag1* ^*+/+*^ *Flt3* ^*fl/fl*^ and *Rag1* ^*cre/+*^ *Flt3* ^*fl/fl*^ mice (*n* = 7 mice per genotype in 2 experiments). (C–D) Mean percentages (±SD of total BM cells) FLT3^*−*^ and FLT3^+^ subsets of (C) CD48^*−*^ CD150^*−*^ ST‐HSCs, CD48^+^ CD150^+^ MPPs and (D) CD48^+^ CD150^*−*^ MPPs and CLPs in 12‐week‐old *Rag1* ^*+/+*^ *Flt3* ^*fl/fl*^ and *Rag1* ^*cre/+*^ *Flt3* ^*fl/fl*^ mice (*n* = 7 mice per genotype in 2 experiments). (E) Mean percentages (±SD of total BM cells) of ProB cells, PreB cells and IgM^+^ B cells in 12‐week‐old *Rag1* ^*+/+*^ *Flt3* ^*fl/fl*^ and *Rag1* ^*cre/+*^ *Flt3* ^*fl/fl*^ mice (*n* = 7 mice per genotype in 2 experiments). (F) Mean percentages (±SD of total thymus cells) of early thymic progenitor (ETP), Double Negative 2 (DN2) and Double Negative 3 (DN3) cells in 12‐weekold adult thymus from *Rag1* ^*+/+*^ *Flt3* ^*fl/fl*^ and *Rag1* ^*cre/+*^ *Flt3* ^*fl/fl*^ mice (*n* = 7 mice per genotype in 2 experiments). *P < 0·05; **P < 0·01; ***P < 0·001; ns, not significant.

Previously reported reductions in the earliest B‐ and T‐lymphoid progenitors in conventional *Flt3* knockout mice could potentially be secondary to reductions in high FLT3‐expressing LMPPs and/or CLPs rather than reflecting a specific and direct role of FLT3 in downstream B‐ and T‐cell committed progenitors. To more specifically investigate the potential direct role of FLT3 down‐stream of adult LMPPs and in already lymphoid‐programmed progenitors, we crossed *Flt3* ^*fl/fl*^ and *Rag1* ^*cre/+*^ mice, to exclusively target loss of FLT3 function to cells already expressing high levels of *Rag1* (McCormack *et al*, 2003). Importantly, and in agreement with only a fraction of adult LMPPs expressing *Rag1* and at very low levels (Adolfsson *et al*, 2005; Mansson *et al*, 2007, 2010; Luc *et al*, 2012), we observed no change in FLT3 cell surface expression on LMPPs, whereas a reduced fraction of Lin^*−*^SCA‐1^low^KIT^low^IL7R^+^ CLPs expressed FLT3 (Fig 2A)**.** Despite this**,** not only long‐term (LT)‐HSCs, short‐term (ST)‐HSCs, CD48^+^CD150^+^ MPPs and CD48^+^CD150^*−*^ MPPs, but also total CLP numbers were unaffected in the BM of adult *Rag1* ^*cre/+*^ *Flt3* ^*fl/fl*^ mice (Fig 2B; Figure S3A), unlike in adult *Vav1* ^*cre/+*^ *Flt3* ^*fl/fl*^ mice (Figure S1B–C). The proportion of CD48^*−*^CD150^*−*^ ST‐HSCs, CD48^+^CD150^+^ MPPs and CD48^+^CD150^*−*^ MPPs, expressing FLT3 were not altered (Fig 2C–D). Notably, although the total numbers of CLPs were unaffected, the FLT3^+^ fraction of CLPs in *Rag1* ^*cre/+*^ *Flt3* ^*fl/fl*^ mice was significantly reduced with a corresponding increase in FLT3^*−*^ CLPs (Fig 2C–D), compatible with a fraction of CLPs being sustained at normal levels although deleted for FLT3 expression. Notably, even if LMPP and CLP numbers in adult *Rag1* ^*cre/+*^ *Flt3* ^*fl/fl*^ mice were unaffected, ProB, PreB and IgM^+^ B cells in the BM (Fig 2E) as well as ETPs in the thymus were significantly reduced (Fig 2F; Figure S3B), to a similar degree as in *Vav1* ^*cre/+*^ *Flt3* ^*fl/fl*^ mice (Figure S1D–E), establishing a strict requirement for FLT3, independently of LMPPs and CLPs, and after initiation of lymphoid‐restricted gene expression in adult haematopoiesis.

**Figure 3:**
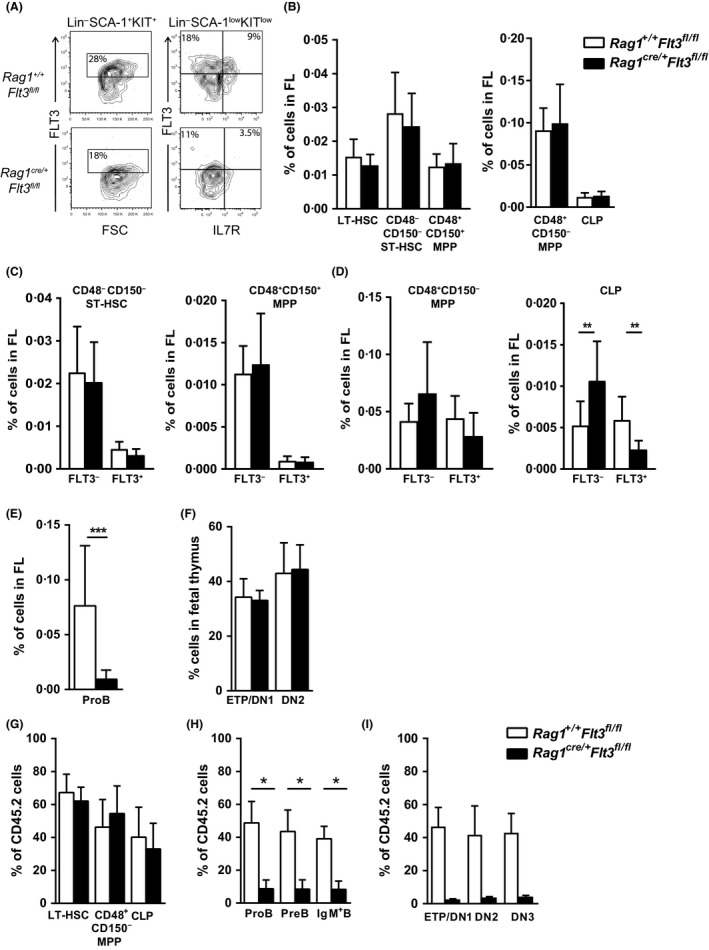
Role of FLT3 in fetal lymphoid‐committed progenitors. (A) Representative fluorescence‐activated cell sorting (FACS)profiles showing FLT3 surface expression on Lin^−^SCA‐1^+^KIT^+^ cells and Lin^−^SCA‐1^low^KIT^low^ cells in *Rag1* ^*+/+*^ *Flt3* ^*fl/fl*^ compared to *Rag1* ^*cre/+*^ *Flt3* ^*fl/fl*^ E14.5 fetal liver (FL) cells (numbers represent mean percentages of 8–11 embryos per genotype). (B) Mean percentages (±SD of total FL cells) of CD48^*−*^ CD150^+^ long‐term haematopoietic stem cells (LT‐HSCs), CD48^*−*^ CD150^*−*^ short‐term (ST)‐HSCs, CD48^+^ CD150^+^ multipotent progenitors (MPPs), CD48^+^ CD150^*−*^ MPPs and common lymphoid progenitors (CLPs) in E14.5 *Rag1* ^*+/+*^ *Flt3* ^*fl/fl*^ and *Rag1* ^*cre/+*^ *Flt3* ^*fl/fl*^ embryos (*n* = 8–11 embryos per genotype in 2 experiments). (C–D) Mean percentages (±SD of total FL cells) FLT3^*−*^ and FLT3^+^ subsets of (C) CD48^*−*^ CD150^*−*^ ST‐HSCs, CD48^+^ CD150^+^ MPPs and (D) CD48^+^ CD150^*−*^ MPPs and CLPs in E14.5 *Rag1* ^*+/+*^ *Flt3* ^*fl/fl*^ and *Rag1* ^*cre/+*^ *Flt3* ^*fl/fl*^ embryos (*n* = 8–11 embryos per genotype in 2 experiments). (E) Mean percentages (±SD of total FL cells) of ProB cells in E14.5 *Rag1* ^*+/+*^ *Flt3* ^*fl/fl*^ and *Rag1* ^*cre/+*^ *Flt3* ^*fl/fl*^ embryos (*n* = 8–11 embryos per genotype in 2 experiments). (F) Mean percentages (±SD of total fetal thymus cells) of early thymic progenitor (ETP) and Double Negative 2 (DN2) cells in E14.5 *Rag1* ^*+/+*^ *Flt3* ^*fl/fl*^ and *Rag1* ^*cre/+*^ *Flt3* ^*fl/fl*^ embryos (*n* = 4–7 embryos per genotype in 1 experiment). (G–I) Mean percentages (±SD) contribution of CD45.2 cells to (G) LT‐HSCs, CD48^+^ CD150^*−*^ MPPs and CLPs, (H) ProB cells, PreB cells, and IgM^+^ B cells in bone marrow and (I) ETP, DN2 and Double Negative 3 (DN3) cells in thymus of lethally‐irradiated CD45.1 mice transplanted with 2 × 10^6^ cells unfractionated E14.5 FL cells from *Rag1* ^*+/+*^ *Flt3* ^*fl/fl*^ (CD45.2) or *Rag1* ^*cre/+*^ *Flt3* ^*fl/fl*^ (CD45.2) mice (*n* = 5 per genotype) together with 2 × 10^6^ cells unfractionated E14.5 FL competitor cells from wild type CD45.1 embryos, analysed 8 weeks post‐transplantation. *P < 0·05; **P < 0·01; ***P < 0·001.

Previous studies have suggested that the cytokine requirement might be distinct for fetal and adult lymphoid progenitors (Carvalho *et al*, 2001; Vosshenrich *et al*, 2003; Hesslein *et al*, 2006; Beaudin *et al*, 2016; Zriwil *et al*, 2016). Moreover, LMPPs in the FL express higher levels of lymphoid genes, including *Rag1,* when compared to their adult counterparts (Boiers *et al*, 2013). We therefore next investigated the impact of *Flt3* deletion in the E14.5 FL of *Rag1* ^*cre/+*^ *Flt3* ^*fl/fl*^ embryos. In agreement with their higher *Rag1* expression (Boiers *et al*, 2013), we observed a slight reduction in LSK cells expressing FLT3 and a more distinct and significant reduction on Lin^−^SCA‐1^low^KIT^low^IL‐7R^+^ CLPs (Fig 3A). Despite this, not only CD48^*−*^CD150^+^ LT‐HSCs, CD48^*−*^CD150^*−*^ ST‐HSCs and CD48^+^CD150^+^ MPPs, but also CD48^+^CD150^*−*^ MPPs and CLPs were unaffected in the E14.5 *Rag1* ^*cre/+*^ *Flt3* ^*fl/fl*^ FL (Fig 3B). Whereas the FLT3^+^ and FLT3^*−*^ fractions of the different LSK HSC and MPP fractions were unaffected in the *Rag1* ^*cre/+*^ *Flt3* ^*fl/fl*^ FL, FLT3^+^ CLPs were reduced and FLT3^*−*^ CLPs correspondingly increased (Fig 3C–D), similar to what was observed in the adult BM. Notably, the earliest ProB cell progenitors emerging in the FL at this stage, were reduced by almost 90%, demonstrating a direct and critical role of FLT3 in fetal B cell progenitors (Fig 3E, Figure S4A–B). In contrast, the earliest thymic progenitors in the E14.5 thymus were unaffected in *Rag1* ^*cre/+*^ *Flt3* ^*fl/fl*^ embryos (Fig 3F, Figure S4C). Even when E14.5 CD45.2 *Rag1* ^*cre/+*^ *Flt3* ^*fl/fl*^ FL cells were competitively transplanted into lethally‐irradiated adult wild‐type (WT) CD45.1 recipients, we observed no impact of *Flt3* deficiency on the earliest LMPP and CLP progenitor compartments (Fig 3G), while early B‐ and T‐cell progenitors were distinctly reduced (Fig 3H–I, Figure S4D–F), again supporting a distinct role of FLT3 in early lymphoid progenitors subsequent to the initiation of lymphoid lineage programming.

### Role of FLT3 in maintenance of distinct B cell subsets

**Figure 4:**
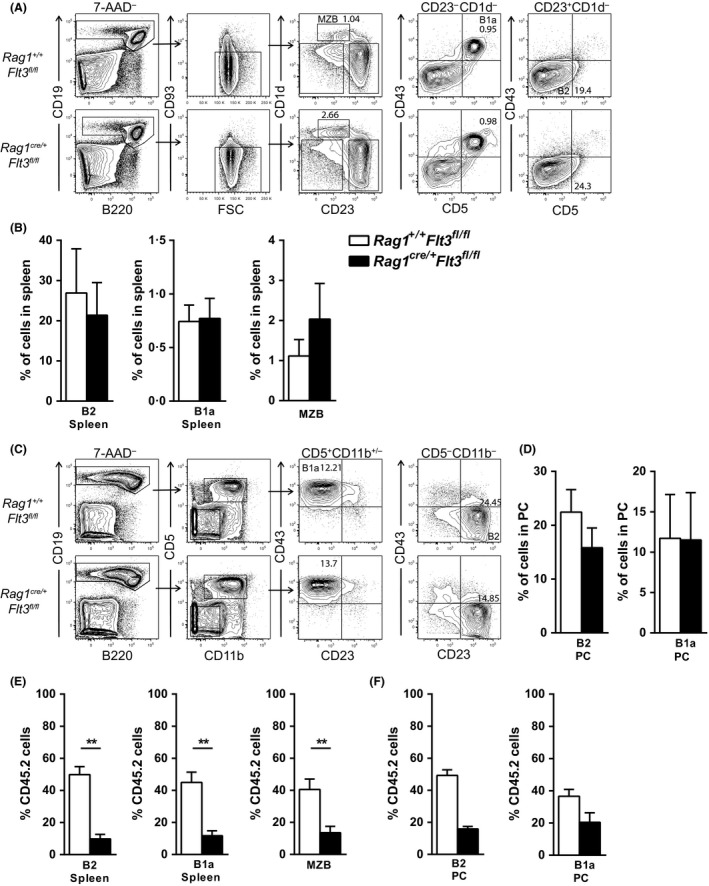
Role of FLT3 in generation of distinct subsets of B cells. (A) Representative fluorescence‐activated cell sorting (FACS)profiles of Follicular B2 cells (CD19^+^ CD93^*−*^ CD5^*−*^ CD43^*−*^ CD23^+^ CD1d^*−*^), Marginal Zone B cells (MZB: CD19^+^ CD93^*−*^ CD23‐CD1d^+^) and B1a cells (CD19^+^ CD93^*−*^ CD5^+^ CD43^+^ CD23^*−*^ CD1d^*−*^) in spleen in 12‐week‐old *Rag1* ^*+/+*^ *Flt3* ^*fl/fl*^ and *Rag1* ^*cre/+*^ *Flt3* ^*fl/fl*^ mice (numbers in gates represent percentages of total spleen cells). (B) Mean percentages (±SD of total spleen cells) of follicular B2, MZB and B1a cells in 12‐week‐old *Rag1* ^*+/+*^ *Flt3* ^*fl/fl*^ and *Rag1* ^*cre/+*^ *Flt3* ^*fl/fl*^ mice (*n* = 6 mice per genotype in 2 experiments). (C) Representative FACS profiles of B2 cells (CD19^+^ CD5^*−*^ CD43^*−*^ CD23^+^ CD11b^*−*^) and B1a cells (CD19^+^ CD5^+^ CD43^+^ CD23^*−*^) in the peritoneal cavity (PC) of 12‐week‐old *Rag1* ^*+/+*^ *Flt3* ^*fl/fl*^ and *Rag1* ^*cre/+*^ *Flt3* ^*fl/fl*^ mice (numbers in gates represent percentages of total PC cells). (D) Mean percentages (±SD of total PC cells) B2 cells and B1a cells of 12‐week‐old *Rag1* ^*+/+*^ *Flt3* ^*fl/fl*^ and *Rag1* ^*cre/+*^ *Flt3* ^*fl/fl*^ mice (*n* = 6 mice per genotype in 2 experiments). (E–F) Mean percentages (±SD) contribution of CD45.2 cells to (E) follicular B2 cells, MZB cells and B1a cells in the spleen and to (F) B2 cells and B1a cells in the PC of CD45.1 wild type (WT) mice transplanted with 2 × 10^6^ unfractionated E14.5 FL cells from *Rag1* ^*+/+*^ *Flt3* ^*fl/fl*^ (CD45.2) or *Rag1* ^*cre/+*^ *Flt3* ^*fl/fl*^ (CD45.2) mice (*n* = 2–5 per genotype) together with 2 × 10^6^ cells unfractionated E14.5 FL WT CD45.1 competitor cells, analysed 8 weeks post‐transplantation.

As we observed a strong impact of lymphoid‐restricted deletion of FLT3 on B‐progenitor cell maintenance in the embryo as well as in adult haematopoiesis, we next investigated to what degree the generation and maintenance of the preferentially fetal‐derived mature B1a and Marginal Zone B (MZB) cells (Hardy & Hayakawa, 1991; Kantor *et al*, 1992; Yoshimoto *et al*, 2011) as well as conventional B2 cells which are also produced during adult haematopoiesis (Hao & Rajewsky, 2001) are dependent on intact FLT3 function. At steady state, follicular B2 cells (CD19^+^CD93^*−*^CD5^*−*^CD43^*−*^CD23^+^CD1d^*−*^) were unaffected in the spleen and peritoneal cavity (CD19^+^CD5^*−*^CD43^*−*^CD23^+^CD11b^*−*^) of adult *Rag1* ^*cre/+*^ *Flt3* ^*fl/fl*^ mice, as were B1a (CD19^+^CD93^*−*^CD5^+^CD43^+^CD23^*−*^CD1d^*−*^) and MZB (CD19^+^CD93^*−*^CD23‐CD1d^+^) cells in the spleen and B1a (CD19^+^CD5^+^CD43^+^CD23^*−*^) cells in the peritoneal cavity (Fig 4A–D, Figure S3C). Fetal‐derived B1a cells and MZB cells are long‐lived and possess self‐renewal potential (Hao & Rajewsky, 2001), which could result in a compensatory expansion to correct any reductions due to loss of FLT3 function. To further assess the role of FLT3 in maintenance of B1a and MZB cells, we therefore analysed the spleen and peritoneal cavity of mice transplanted with unfractionated E14.5 FL cells from CD45.2 *Rag1* ^*cre/+*^ *Flt3* ^*fl/fl*^ embryos, together with competitor E14.5 FL WT CD45.1 cells. At 8 weeks following transplantation, we observed an impairment in reconstitution of B1a, MZB as well as B2 cells in the spleen and peritoneal cavity of recipients of *Rag1* ^*cre/+*^ *Flt3* ^*fl/fl*^ FL cells (Fig 4E–F), establishing an important role of FLT3 for the homeostasis of each of these distinct B cell populations from already lymphoid‐restricted progenitors.

## Discussion

Despite the established role of FLT3 in B‐ and T‐lymphopoiesis (Mackarehtschian *et al*, 1995; McKenna *et al*, 2000; Sitnicka *et al*, 2003, 2007; Buza‐Vidas *et al*, 2007), and the involvement of recurrent *FLT3* mutations in acute B‐ and T‐cell progenitor leukaemia (Carow *et al*, 1996; Gilliland & Griffin, 2002; Armstrong *et al*, 2004; Neumann *et al*, 2013), it has remained unclear whether lymphoid‐restricted progenitors are directly dependent on FLT3. This is particularly relevant because FLT3 expression is highest and critically important on the earliest adult lympho‐myeloid LMPPs, of which only a fraction express low levels of *Rag1* (Adolfsson *et al*, 2005), whereas only the very earliest T‐ and B‐cell progenitors express FLT3 (Wasserman *et al*, 1995; Mansson *et al*, 2010; Luc *et al*, 2012). Herein, we developed a *Flt3* conditional knockout mouse model to specifically investigate a potential requirement for FLT3 in progenitors already programmed for lymphopoiesis, as determined by high *Rag1* expression, as well as potentially distinct roles in fetal and adult lymphopoiesis.

Our studies confirmed that while having no role in the regulation of HSCs, FLT3 is important for sustaining normal numbers of LMPPs in adult BM as well as ETPs in the adult thymus. In contrast, CLPs were only minimally affected when induced to loose FLT3 expression in adult mice, establishing that LMPPs are more dependent on intact FLT3 expression and function than CLPs in adult steady‐state haematopoiesis. Notably, B‐cell progenitor numbers were also unaffected upon inducible pan‐haematopoietic deletion of FLT3 in adult mice. Nevertheless, molecular analysis of B‐cell progenitors demonstrated that *Flt3‐*deleted adult progenitors, such as LMPPs or CLPs, have a considerable competitive disadvantage in producing B‐cell progenitors compared to the rare progenitors escaping *Flt3* deletion in our model. In agreement with this observation, competitive Mx1Cre adult BM transplantation experiments demonstrated as soon as 4 weeks post‐transplantation that not only the generation of CD48^+^CD150^*−*^MPPs (representing predominantly LMPPs) and T‐cell progenitors, but also CLPs, B‐cell progenitors, and mature B cells were significantly impaired from FLT3‐deleted BM cells.

To address whether FLT3 plays a direct role in the regulation of adult lymphoid‐restricted progenitor maintenance, rather than in their generation from LMPPs, we specifically deleted *Flt3* through *Rag1* ^*cre/+*^‐induced recombination (McCormack *et al*, 2003). As expected, due to their low levels of *Rag1* expression, FLT3 expression was unaffected on LMPPs as were LMPP numbers in adult BM. Notably, whereas FLT3 expression was deleted in approximately 50% of CLPs, CLP number was also unaffected in the BM of adult *Rag1* ^*cre/+*^ *Flt3* ^*fl/fl*^ mice. This allowed for the specific establishment of a distinct role of FLT3 in direct regulation of early B‐ and T‐cell progenitors independently of the role of FLT3 in regulation of earlier LMPPs and CLPs, because the earliest BM B‐cell progenitors and thymic T‐cell progenitors were distinctly reduced in adult *Rag1* ^*cre/+*^ *Flt3* ^*fl/fl*^ mice, in the absence of an impact on LMPPs and CLPs. This is of considerable significance, given that FLT3 expression in the B and T cell lineages is restricted to the very earliest B‐ and T‐cell progenitors, pre‐proB cells (Wasserman *et al*, 1995) and ETPs (Luc *et al*, 2012), respectively. Importantly, these progenitors are likely to also be key cellular targets for recurrent *FLT3* driver mutations in patients with acute B‐cell progenitor and ETP leukaemia (Carow *et al*, 1996; Armstrong *et al*, 2004; Neumann *et al*, 2013). Our findings therefore highlight the potential benefit of targeting these leukaemias with clinical FLT3 inhibitors (Annesley & Brown, 2014).

Also in the liver of *Rag1* ^*cre/+*^ *Flt3* ^*fl/fl*^ embryos, we observed a dramatic reduction in early B‐cell progenitors in the absence of a significant reduction of fetal LMPPs and CLPs, demonstrating a distinct and prominent requirement for FLT3 in fetal B‐lymphoid progenitors, again independently of a role in earlier progenitors. Interestingly, distinct from adult haematopoiesis, E14.5 fetal thymic progenitor homeostasis was not affected in *Rag1* ^*cre/+*^ *Flt3* ^*fl/fl*^ mice, despite FLT3 being expressed on fetal ETPs (Luis *et al*, 2016). However, at 4 weeks after competitive transplantation of *Rag1* ^*cre/+*^ *Flt3* ^*fl/fl*^ FL cells into adult recipients, while CD48^+^CD150^*−*^ MPPs and CLPs remained unaffected, an impaired generation of not only B cell progenitors but also early thymocyte progenitors could be readily detected from *Rag1* ^*cre/+*^ *Flt3* ^*fl/fl*^ FL cells. The distinct thymocyte phenotype observed upon transplantation of E14.5 *Rag1* ^*cre/+*^ *Flt3* ^*fl/fl*^ FL cells into adult recipients but not in the *Rag1* ^*cre/+*^ *Flt3* ^*fl/fl*^ E14.5 fetal thymus, combined with a similar early thymocyte defect observed in unperturbed adult *Rag1* ^*cre/+*^ *Flt3* ^*fl/fl*^ mice, suggest a distinct requirement for FLT3 signalling in adult but not fetal thymopoiesis, potentially explained by distinct differences in the fetal and adult thymic environment. In contrast, fetal and adult B‐lymphopoiesis are both dependent on intact FLT3 expression and function.

Despite its very restricted expression, our studies demonstrate that FLT3 plays an important and distinct role in the direct regulation of early B cell‐restricted progenitors in FL as well as in adult BM. In further agreement with its crucial role in B‐lymphopoiesis, our studies of *Rag1* ^*cre/+*^ *Flt3* ^*fl/fl*^ mice establish a critical role for FLT3 in the maintenance of fetal‐derived B1a and MZB cells (Hardy & Hayakawa, 1991; Kantor *et al*, 1992; Yoshimoto *et al*, 2011) as well as conventional B2 cells (Hao & Rajewsky, 2001). Previous studies have shown that the important role of FLT3 in fetal B1 and B2 lymphopoiesis is even more evident in the absence of interleukin 7 (IL7). Specifically, in the absence of the IL7 ligand and FLT3 ligand, fetal and adult B lymphopoiesis is almost entirely lost, suggesting that thymic stromal lymphoprotein (TSLP), also acting through the IL7 receptor, is unable to rescue B cell development in the absence of FLT3 ligand and IL7 (Sitnicka *et al*, 2003; Jensen *et al*, 2008).

In conclusion, through lymphoid‐restricted and ontogeny‐specific deletion of FLT3 expression and function, we establish that the important role of FLT3 in fetal and adult B‐ and T‐lymphopoiesis is, in fact, not primarily explained by its role in regulation of LMPPs and CLPs, but rather by a direct and more prominent role in the regulation of the very earliest *Rag1* expressing B‐ and T‐ cell progenitors, which are likely to be primary cellular targets for recurrent FLT3 mutations in clinically distinct B‐ and T‐cell progenitor cell leukaemia.

## Author contributions

AZ, ES and SEWJ designed and conceptualized the overall research and analysed the data. CN designed and supervised the generation of the *Flt3* conditional knockout targeting construct and targeting of ES cells. AZ performed the experiments. TAK performed B1 cell analysis experiments. LW provided expertise in the animal work. CB contributed to the design, analysis of experiments, data analysis and writing of the manuscript. JY contributed with expert advice and input on B cell development. AZ, ES and SEWJ wrote the manuscript. All authors read and approved the submitted manuscript.

## Declaration of interest

The authors declare no competing financial interests.

## Supporting information


**Figure** **S1**. Provides data validating the model.
**Figure** **S2**. Provides data that extends the findings in Fig 1.
**Figure** **S3**. Provides data that extends the findings in Figs 2, 3 and 4.
**Figure** **S4**. Provides data that extends the findings in Fig 3.
**Table** **S1**. Provides the list of antibodies used in the study.
