# Supplementary material for: Direct role of FLT3 in regulation of early lymphoid progenitors
Source: Br J Haematol. 2018 Sep 14;183(4):588–600. doi: 10.1111/bjh.15578 (PMC6492191; doi:10.1111/bjh.15578)
Supplement: Supplementary file 1 — Figure S1. Provides data validating the model. Figure S2. Provides data that extends the findings in Fig 1. Figure S3. Provides data that extends the findings in Figs 2, 3 and 4. Figure S4. Provides data that extends the findings in Fig 3. Table S1. Provides the list of antibodies used in the study. [file BJH-183-588-s001.docx]

| **Antibody conjugate** | **Clone** | **Supplier** | **Application** |
| --- | --- | --- | --- |
| B220 APC-Cy7 | RA3-6B2 | Biolegend | B cells staining in PC and in spleen |
| B220 FITC | RA3-6B2 | BD | Lineage cocktail for HSC, LMPP in BM and FL and for T cell progenitors in thymus |
| B220 PE-TxR | RA3-6B2 | BD | B cell progenitors staining in BM and FL |
| CD11b BV605 | M1/70 | BD | B cells staining in PC |
| CD11c FITC | HL3 | BD | Lineage cocktail for T cell progenitors in thymus |
| CD150 PECy7 | TC15-12F12.2 | Biolegend | HSC and LMPP staining in BM and FL |
| CD19 APC | ID3 | BD | B cells staining in PC |
| CD19 BV786 | ID3 | BD | B cells staining in spleen |
| CD19 eF450 | 1D3 | eBioscience | B cell progenitors staining in BM and FL |
| CD1d PercPCy5.5 | B1B | eBioscience | B cells staining in spleen |
| CD23 PECy7 | B3B4 | Biolegend | B cells staining in PC and in spleen |
| CD24 PE | 30-F1 | eBioscience | B cell progenitors staining in BM and FL |
| CD25 PercPCy5.5 | PC61 | BD | T cell progenitors in thymus |
| CD3e FITC | 145-2C11 | BD | Lineage cocktail for HSC, LMPP and B cell progenitors in BM and FL |
| CD4 AF700 | GK1.6 | Biolegend | T cell progenitors in thymus |
| CD4 FITC | H129.19 | BD | Lineage cocktail for HSC, LMPP and B cell progenitors in BM |
| CD43 APC | S7 | BD | B cell progenitors staining in BM and FL |
| CD43 FITC | S7 | BD | B cells staining in PC and in spleen |
| CD45.1 AF700 | A20 | Biolegend | Transplantation staining |
| CD45.1 Biotin | A20 | BD | Transplantation staining |
| CD45.2 PE | 104 | BD | Transplantation staining |
| CD45.2 PercPCy5.5 | 104 | BD | Transplantation staining |
| CD48 APC | HM45-1 | Biolegend | HSC and LMPP staining in BM and FL |
| CD5 BV421 | 53-7.3 | BD | B cells staining in PC and in spleen |
| CD8a FITC | 53-6.7 | BD | Lineage cocktail for HSC, LMPP and B cell progenitors in BM and FL |
| CD8a PB | 53-6.7 | BD | T cell progenitors in thymus |
| CD93 APC | AA4.1 | Biolegend | B cells staining in spleen |
| CD93 PECy7 | AA4.1 | eBioscience | B cell progenitors staining in BM and FL |
| cKIT APC-eF780 | 2B8 | eBioscience | HSC, LMPP, CLP staining in BM and FL and T cell progenitors in thymus |
| FLT3 PE | A2F10.1 | BD | HSC, LMPP, CLP staining in BM and FL |
| GR-1 FITC | RB6-8C5 | BD | Lineage cocktail for HSC, LMPP, B cell progenitors in BM and FL and for T cell progenitors in thymus |
| IgM APC-Cy7 | II/41 | eBioscience | B cell progenitors staining in BM |
| IL7R PETxR | SB/199 | BD | HSC, LMPP, CLP staining in BM and FL |
| Ly6C FITC | AL-21 | BD | Lineage cocktail for B cell progenitors in BM and FL |
| MAC1 FITC | M1/70 | BD | Lineage cocktail for HSC, LMPP, B cell progenitors in BM and for T cell progenitors in thymus |
| NK1.1 FITC | PK136 | BD | Lineage cocktail for HSC, LMPP, B cell progenitors in BM and FL and for T cell progenitors in thymus |
| SCA-1 PB | E13.161.7 | Biolegend | HSC, LMPP, CLP staining in BM and FL |
| TER-119 FITC | Ter-119 | BD | Lineage cocktail for HSC, LMPP, B cell progenitors in BM and FL and for T cell progenitors in thymus |

**Supplemental Table 1 (related to Methods and Materials). Antibodies used for flow cytometry and FACS staining**


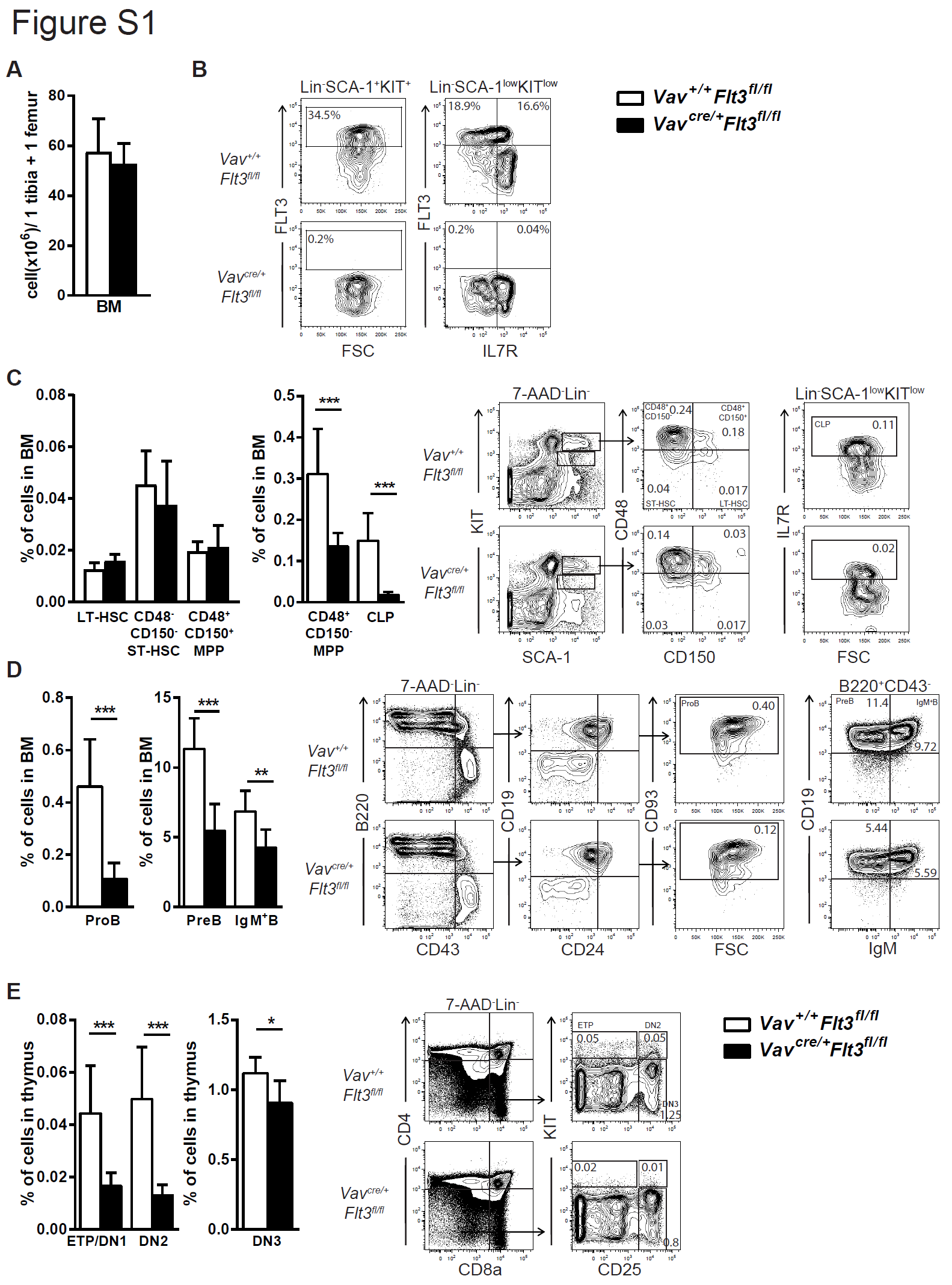


**Supplemental Fig 1. Validation of Flt3 deletion by Vav-cre targeted excision.**

(**A**) Mean (± SD) total BM cells in 12 week old *Vav1^+/+^Flt3^fl/fl^* and *Vav1^cre/+^Flt3^fl/fl^* mice (n=8 mice per genotype in 4 experiments). (**B**) Representative FACS profiles showing FLT3 surface expression on Lin^-^ SCA-1^+^KIT^+^ cells and Lin^-^SCA-1^low^KIT^low^ cells in *Vav1^+/+^Flt3^fl/fl^* compared to *Vav1^cre/+^Flt3^fl/fl^* BM (numbers represent mean percentages of 8 mice per genotype). Gates for FLT3 expression were set using LT-HSCs as a negative internal reference population (IRP). (**C**) (Left) Mean percentages (± SD, of total BM cells) and (right) representative FACS profiles of LT-HSCs (Lin^-^SCA-1^+^KIT^+^CD48^-^CD150^+^), CD48^-^CD150^-^ ST-HSCs (Lin^-^SCA-1^+^KIT^+^CD48^-^CD150^-^), CD48^+^CD150^+^ MPPs (Lin^-^SCA-1^+^KIT^+^CD48^+^CD150^+^), CD48^+^CD150^-^ MPPs (Lin^-^SCA-1^+^KIT^+^CD48^+^CD150^-^) and CLPs (Lin^-^SCA-1^low^KIT^low^IL7R^+^) in 12 week old *Vav1^+/+^Flt3^fl/fl^* and *Vav1^cre/+^Flt3^fl/fl^* mice (n=8 mice per genotype in 4 experiments, numbers in gates represent percentages of total BM cells). (**D**) (Left) Mean percentages (± SD, of total BM cells) and (right) representative FACS profiles of ProB cells (Lin^-^B220^+^CD43^+^CD19^+^CD24^+^CD93^+^), PreB cells (Lin^-^B220^+^CD43^-^CD19^+^IgM^-^) and IgM^+^ B cells (Lin^-^B220^+^CD43^-^CD19^+^IgM^+^) in 12 week old *Vav1^+/+^Flt3^fl/fl^* and *Vav1^cre/+^Flt3^fl/fl^* mice (n=8 mice per genotype in 4 experiments, numbers in gates represent percentages of total BM cells. (**E**) (Left) Mean percentages (± SD of total thymocytes) and (right) representative FACS profiles of ETPs (Lin^-^CD4^-^CD8a^-^KIT^+^CD25^-^), DN2s (Lin^-^CD4^-^CD8a^-^KIT^+^CD25^+^) and DN3s (Lin^-^CD4^-^CD8a^-^KIT^-^CD25^+^) in 12 weeks old *Vav1^+/+^Flt3^fl/fl^* and *Vav1^cre/+^Flt3^fl/fl^* mice (n=8 mice per genotype in 4 experiments, numbers in gates represent percentages of total BM cells).

*P<.05; **P<.01; ***P<.001


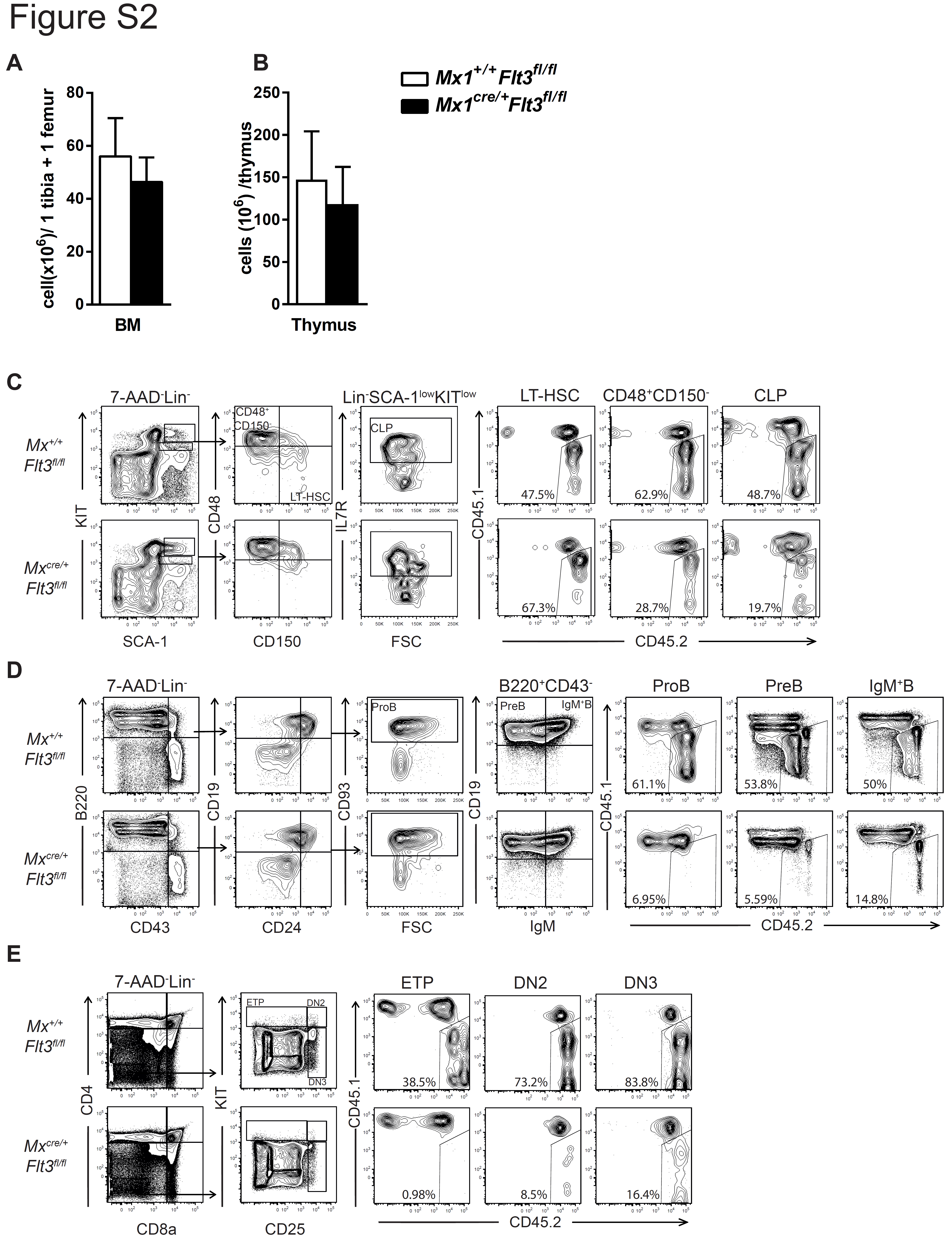


**Supplemental Fig 2 (related to Fig 1).**

Mean (± SD) total (**A**) BM cells and (**B**) thymocytes in 12 week old *Mx1^+/+^Flt3^fl/fl^* and *Mx1^cre/+^Flt3^fl/fl^* mice (n=6-8 mice per genotype in 3 experiments) 4 weeks after pIpC injection. (**C-E**) Representative FACS profiles of CD45.2/CD45.1 distribution within (**C**) LT-HSCs (Lin^-^ SCA-1^+^KIT^+^CD48^-^CD150^+^), CD48^+^CD150^-^ MPPs (Lin^-^ SCA-1^+^KIT^+^CD48^+^CD150^-^) and CLPs (Lin^-^SCA-1^low^KIT^low^IL7R^+^), (**D**) ProB (Lin^-^B220^+^CD43^+^CD19^+^CD24^+^CD93^+^) cells, PreB (Lin^-^B220^+^CD43^-^CD19^+^IgM^-^) cells, and IgM^+^ B (Lin^-^B220^+^CD43^-^CD19^+^IgM^+^) cells in BM and (**E**) ETP (Lin^-^CD4^-^CD8a^-^KIT^+^CD25^-^), DN2 (Lin^-^CD4^-^CD8a^-^KIT^+^CD25^+^) and DN3 (Lin^-^CD4^-^CD8a^-^KIT^-^CD25^+^) cells in thymus of lethally irradiated CD45.1 mice transplanted with 2x10^6^ cells unfractionated BM cells from Mx1^+/+^Flt3^fl/fl^ (CD45.2) or Mx1^cre/+^Flt3^fl/fl^ (CD45.2) mice together with 2x10^6^ cells unfractionated BM competitor cells from WT CD45.1 mice, analysed 8 weeks post-transplantation and 4 weeks after pIpC injection (numbers in gates represent percentages of CD45.2 cells within the indicated cell populations).

**Supplemental Fig 3 (related to Fig 2,3 and 4). Tissue cellularities.**

Mean (± SD) number of total (**A**) BM cells, (**B**) thymocytes and (**C**) splenocytes in 12 week old *Rag1^+/+^Flt3^fl/fl^* and *Rag1^cre/+^Flt3^fl/fl^* mice (n=6-8 mice per genotype in 2 experiments).

**P<.01





**Supplemental Fig 4 (related to Fig 3).**

(**A**) Mean (± SD) number of total FL cells in E14.5 *Rag1^+/+^Flt3^fl/fl^* and *Rag1^cre/+^Flt3^fl/fl^* embryos (n=8-11 embryos per genotype in 2 experiments). (**B-C**) Representative FACS profiles of (**B**) ProB (Lin^-^B220^+^CD43^+^CD19^+^CD24^+^CD93^+^) cells in FL and (**C**) ETP (Lin^-^CD4^-^CD8a^-^KIT^+^CD25^-^) and DN2 (Lin^-^CD4^-^CD8a^-^KIT^+^CD25^+^) cells in fetal thymus of E14.5 *Rag1^+/+^Flt3^fl/fl^* and *Rag1^cre/+^Flt3^fl/fl^* embryos (numbers in gates represent percentages of total BM cells). (**D-F**) Representative FACS profiles of CD45.2/CD45.1 distribution within (**D**) LT-HSCs (Lin^-^ SCA-1^+^KIT^+^CD48^-^CD150^+^), CD48^+^CD150^-^ MPPs (Lin^-^ SCA-1^+^KIT^+^CD48^+^CD150^-^) and CLPs (Lin^-^SCA-1^low^KITl^ow^IL7R^+^), (**E**) ProB cells, PreB (Lin^-^B220^+^CD43^-^CD19^+^IgM^-^) cells, and IgM^+^ B (Lin^-^B220^+^CD43^-^CD19^+^IgM^+^) cells in BM and (**F**) ETP, DN2 and DN3 (Lin^-^CD4^-^CD8a^-^KIT^-^CD25^+^) cells in thymus of lethally irradiated CD45.1 mice transplanted with 2x10^6^ cells unfractionated E14.5 FL cells from *Rag1^+/+^Flt3^fl/fl^* (CD45.2) or *Rag1^cre/+^Flt3^fl/fl^* (CD45.2) embryos together with 2x10^6^ cells unfractionated E14.5 FL competitor cells from WT CD45.1 embryos, analysed 8 weeks post-transplantation (numbers in the gates represent percentages of CD45.2 cells within the indicated cell population).
